# Non-invasive high frequency oscillatory ventilation inhibiting respiratory motion in healthy volunteers

**DOI:** 10.1038/s41598-022-27288-3

**Published:** 2022-12-30

**Authors:** Yanshan Zhang, Xiaojun Li, Yihe Zhang, Yancheng Ye, Yee-Min Jen, Xin Pan, Xiaowei Li, Tianyan Qin, Pengqing Li, Caixia Lv, Ying Qi, Xin Wang, Yuling Yang, Tong Ma

**Affiliations:** 1 Heavy Ion Center, Wuwei Cancer Hospital, No. 31 Sanitary Lane, Haizang Road, Wuwei, 733000 Gansu Province China; 2 Department of Radiation Oncology, Yee Zen General Hospital, 30, Yangxing North Rd, Yang Mei District, Tao Yuan City, Taiwan

**Keywords:** Medical research, Oncology

## Abstract

Precision radiotherapy needs to manage organ movements to prevent critical organ injury. The purpose of this study is to examine the feasibility of motion control of the lung by suppressing respiratory motion. The non-invasive high frequency oscillatory ventilation (NIHFOV) is a technique commonly used in the protection of lung for patients with acute lung disease. By using a very high respiratory frequency and a low tidal volume, NIHFOV allows gas exchange, maintains a constant mean airway pressure and minimizes the respiratory movements. We tested healthy volunteers NIHFOV to explore the optimal operational parameter setting and the best possible motion suppression achievable. This study was conducted with the approval of Institutional Review Boards of the Wuwei Cancer hospital (approval number: 2021-39) and carried out in accordance with Declaration of Helsinki. The study comprises two parts. Twenty three healthy volunteers participated in the first part of the study. They had 7 sessions of training with the NIHFOV. The duration of uninterrupted, continuous breathing under the NIHFOV and the optimal operational machine settings were defined. Eight healthy volunteers took part in the second part of the study and underwent 4-dimensional CT (4DCT) scanning with and without NIHFOV. Their respiratory waveform under free breathing (FB) and NIHFOV were recorded. The maximum range of motion of the diaphragm from the two scannings was compared, and the variation of bilateral lung volume was obtained to evaluate the impact of NIHFOV technique on lung volume. The following data were collected: comfort score, transcutaneous partial pressure of oxygen (PtcO_2_), transcutaneous partial pressure of carbon dioxide (PtcCO_2_), and pulse rate. Data with and without NIHFOV were compared to evaluate its safety, physiological impacts and effect of lung movement suppression. All the volunteers completed the training sessions eventlessly, demonstrating a good tolerability of the procedure. The median NIHFOV-on time was 32 min (22–45 min), and the maximum range of motion in the cephalic-caudal direction was significantly reduced on NIHFOV compared with FB (1.8 ± 0.8 cm vs 0.3 ± 0.1 cm, *t* =  − 3.650, *P* = 0.003); the median range of motion was only 0.3 ± 0.1 cm on NIHFOV with a good reproducibility. The variation coefficient under NIHFOV of the right lung volume was 2.4% and the left lung volume was 9.2%. The PtcO_2_ and PtcCO_2_ were constantly monitored during NIHFOV. The medium PtcCO_2_ under NIHFOV increased lightly by 4.1 mmHg (interquartile range [IQR], 4–6 mmHg) compared with FB (*t* = 17.676, *P* < 0.001). No hypercapnia was found, PtcO_2_ increased significantly in all volunteers during NIHFOV (*t* = 25.453, *P* < 0.001). There was no significant difference in pulse rate between the two data sets (*t* = 1.257, *P* = 0.233). NIHFOV is easy to master in healthy volunteers to minimize respiratory movement with good tolerability and reproducibility. It is a feasible approach for lung motion control and could potentially be applied in accurate radiotherapy including carbon-ion radiotherapy through suppression of respiratory movement.

## Introduction

To conduct precision radiotherapy, during which radiation to the adjacent normal organs is minimized, organ movement control is essential. Respiration is the most common and significant cause of organ movement. This respiration-related movement affects all organs of the chest and abdomen, from the esophagus to the prostate^1^. It limits precise irradiation of the tumor, increases the risk of critical organ damage and results in reduced effectiveness of radiotherapy. To deal with the movement of organs, in addition to increasing the margin boundary around the target, methods used to reduce respiratory movement include abdominal compression, gating technique, deep inspiratory breath hold, and real-time tracking technology. All of these methods have some technical challenges and limitations^2^. At present, there is no consensus on how to best manage or compensate respiratory movement.

To our best knowledge, our heavy ion center was the first hospital in China to conduct precision radiotherapy with suppression of respiratory motion under general anesthesia in 2016^3^, and we used this apnea-like technique in intensity-modulated radiotherapy (IMRT) and stereotactic body radiotherapy (SBRT)/stereotactic ablative radiotherapy (SABR). But this technique has the following disadvantages: patients had muscle relaxants under general anesthesia to abolish the respiratory movement, which, apart from the potential risk of a general anesthesia including CO_2_ retention and hypoxaemia, required the presence of an anesthesia team. Our center is equipped with the first home-made carbon ion facility in China, and carbon ion radiotherapy often is delivered with fewer fraction numbers than that in photon radiotherapy due to its physical and biological features. To fully exploit the advantages of carbon ion radiotherapy, we intend to apply the non-invasive high-frequency oscillatory ventilation (NIHFOV) technique to control breathing movement.

The high frequency oscillatory ventilation is a technique commonly used in the protection of lung for patients with acute lung disease who requires mechanical ventilation. By using a very high respiratory frequency and a low tidal volume, NIHFOV allows to maintain a constant mean airway pressure much lower than that with a conventional ventilator^4,5^. We hypothesize that INHFOV can keep the patient well oxygenated while minimizing the respiratory movements, making it a useful option for motion control in radiotherapy. After approval of the Institutional Ethics Committee of Wuwei Cancer Hospital, we underwent a pilot study to examine the feasibility, safety, and efficacy of the NIHFOV. We hereby reported the results.

## Materials and methods

### Participants, the ventilator and 4-dimensional CT simulator

The study conforms to the requirements of GCP, Helsinki Declaration and relevant domestic laws and regulations. The protocol and informed consent have gained approval by the institutional ethical review committee, the register number is 2021-Ethical review-39. All volunteers gave informed consent. Twenty three healthy volunteers received NIHFOV training. Only 8 of the 23 volunteers underwent 4DCT scanning, including 5 males and 3 females, aged 22 to 69 (24.9 ± 2.6) years. The other 15 volunteers did not want to exposure to 4DCT X-ray due to fertility needs, health reasons and other reasons.

**Figure 1:**
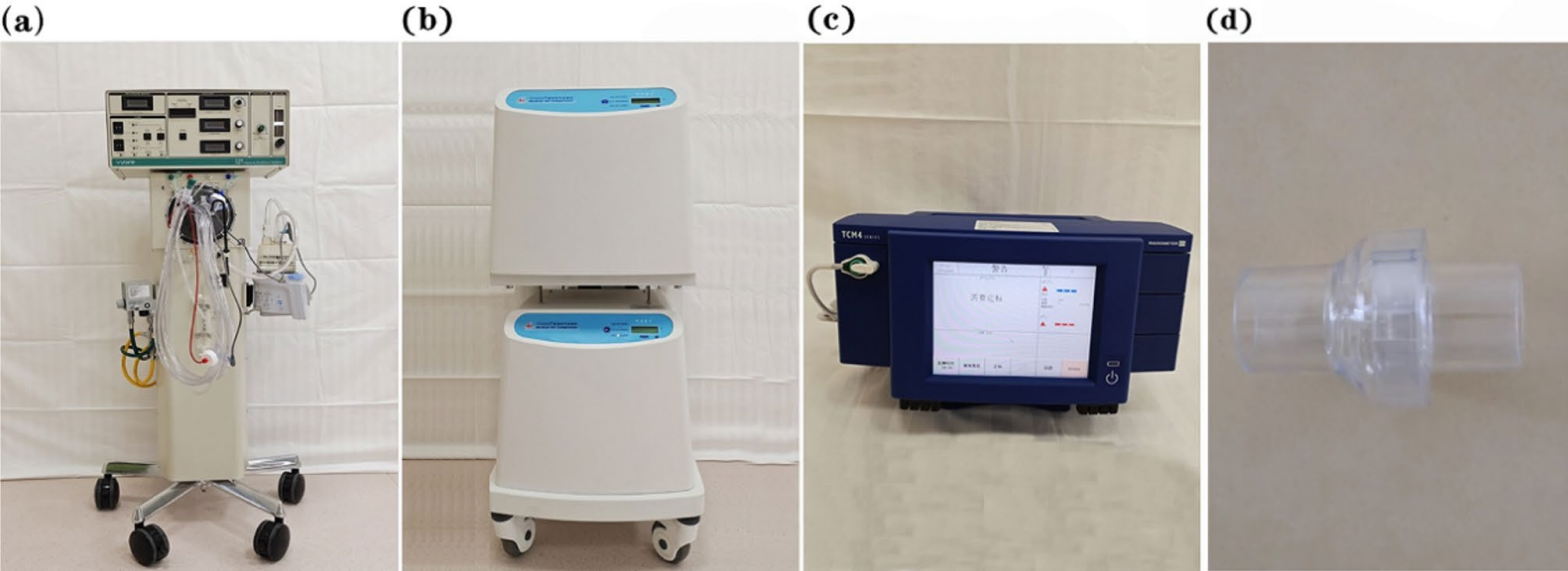
Non-invasive high-frequency oscillatory ventilator (NIHFOV) and its auxillaries. (**a**) High-frequency oscillatory ventilator; (**b**) Medical air compressor; (**c**) Percutaneous partial pressure of oxygen/carbon dioxide monitor; (**d**) leakage valve of non-invasive the ventilator circuit.

**Figure 2:**
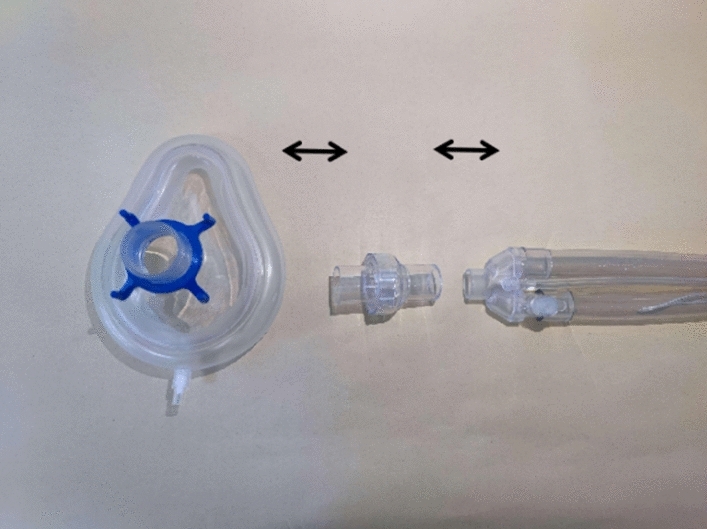
Schematic diagram: connection of mask, leak valve and ventilator tube connection.

**Figure 3:**
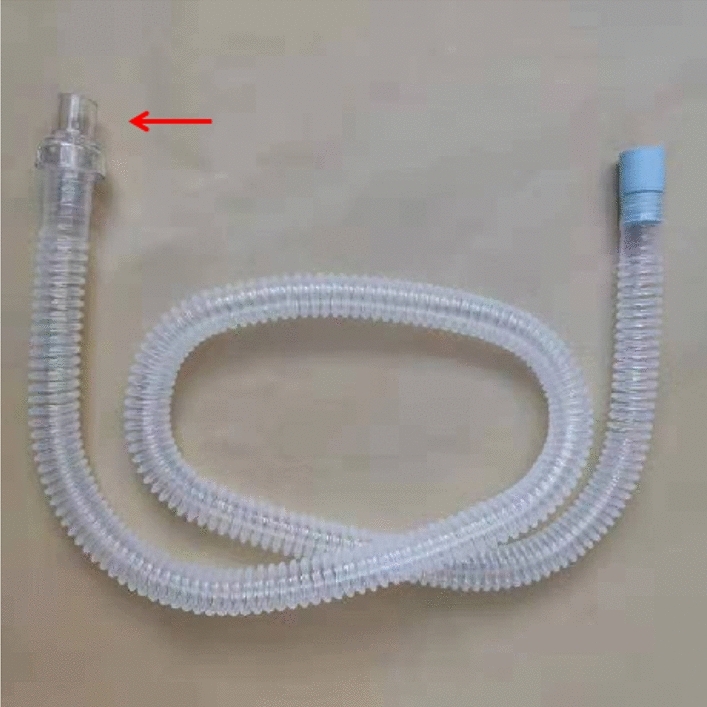
Carina disposable non-invasive tube with a leaking valve (MP00312, Ventstar Carina LeakV, Drager, Canada), in our study, we only cut and use the leaking valve (red arrow).

**Figure 4:**
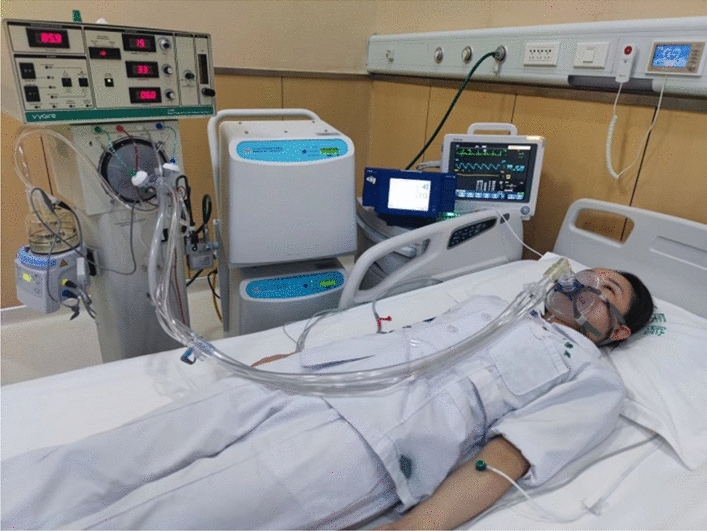
One volunteer is training with NIHFOV.

The set of NIHFOV system comprises the following parts: High-frequency oscillatory ventilator, Model 3100B (CareFusion 3100B HFOV, California, USA) (Fig. 1a); a medical air compressor (TF5000, Beijing Shenlu, China) (Fig. 1b); transcutaneous partial pressure of oxygen/carbon dioxide monitor (TCM Combi M, Radiometer, Denmark) (Fig. 1c); non-invasive ventilation masks; Carina disposable non-invasive tube with a leaking valve (MP00312, Ventstar Carina LeakV, Drager, Canada) (Fig. 1d), bedside multi-parameter ECG monitor. The Fig. 2 shows the Carina disposable non-invasive tube with a leaking valve, in our study, we only cut and use the leaking valve. Figure 3 shows how to connect the mask, leaking valve, ventilator tube connection, Fig. 4 shows one volunteer is training with NIHFOV.

A Siemens Big Bore scanner (SOMATOM Definition AS, Siemens, German) was used to acquire all CT images of free breathing (FB) and NIHFOV. The Anzai system (Anzai Medical, Japan) was used to gather the breathing signals. The Anzai system is the respiratory gating system was developed in collaboration with Tsukuba University Hospital in 1987. It has perfected Respiratory Gating System, in response to demand for faster respiratory gating systems in the radiotherapy field, the sysytem have achieved a respiratory signal output delay of 50 ms or less between detection of respiratory signals and respiratory signal output^6^.

### Study design and NIHFOV procedure

The study included two parts. During the first part, subjects underwent NIHFOV-on breathing training to familiarize with the technique. The goal of this phase was to examine the feasibility and safety of NIHFOV and find the optimal operational settings of the NIHFOV system. All the 23 participants underwent the part one study. The second part of the study was to determine the feasibility of the NIHFOV technique in the inhibition of respiratory movements. Subjects had to undergo two 4DCT scanning, one with free breathing (FB) another one with NIHFOV. Due to the previously mentioned reason, only eight of the 23 volunteers took part in this phase of the study.

Before the first training of part one, volunteers accepted NIHFOV instruction including the working mechanism, safety, and an emphasis of trying to breathe as shallowly as possible to reduce the amplitude of respiration. The subject could stop the procedure any time of the training session when they want to do so. Volunteers wore a noninvasive ventilation mask with a leakage valve, which was then connected to the HFOV machine. This setup allows continuous monitoring of the airway pressure and allows the subject to unplug Ventilator tube from the mask to resume spontaneous breathing at any time during NIHFOV session. NIHFOV breathing training was performed once a day, and the ventilator setting was adjusted according to the tolerance and comfort of the trainee. Although all subjects believed that they can fully adapt to this technology after 3 or 4 times of training, we required each subject to complete 7 times of training for the stability of NIHFOV. A total of 7 training sessions were performed for every participant, and data of each session were recorded. Evaluate the training tolerance of each subject. The training lasted until the subjects fully adapted to the ventilator and complete 7 times of training, and the deep exhalation was less than once per minute, and the NIHFOV-on duration was more than 20 min. After the completion of the seventh training session, the subjects were considered to be well tolerated and the training effect was evaluate satisfactory, 4DCT can be collected. The 8 subjects in the part two had a 4DCT with NIHFOV.

The initial setting of the HFOV were as follows: oxygen concentration 100%, bias flow 40 L/min, respiratory rate (RR) 7 Hz (420/min), percentage of inspiratory time 33%, amplitude 8 cmH_2_O, mean airway pressure (MAP) 10 cm H_2_O (8–12 cm H_2_O), and the airway pressure lower and upper limits alarm threshold value of the ventilator 5 cm H_2_O and 25 cm H_2_O. The Anzai Respiratory gating system was coupled to a 4D-CT simulator to record the amplitude of thoracic movement during respiration under FB. Firstly, 4D-CT of the FB was collected, and then 4D-CT of the NIHFOV-on was collected. Because the chest wall mobility was too small to trigger the Anzai respiratory gated system in NIHFOV-on state, which could not detect the amplitude signals of thoracic respiration, we use the Anzai respiratory simulator induced 4D-CT scan in NIHFOV-on state. Throughout the NIHFOV session, subjects’ transcutaneous partial pressure of oxygen (PtcO_2_) and transcutaneous partial pressure of carbon dioxide (PtcCO_2_) were monitored with a transcutaneous monitoring unit as well as the heart rate (HR) and blood pressure with a multi-parameter ECG monitor at the bedside.

### Study endpoints

NIHFOV-on tolerability was evaluated by a comfort scale using the following analog scale: 1 = unacceptable, 2 = poor, 3 = good, 4 = very good, 5 = excellent.

NIHFOV-on duration refers to the uninterrupted time from the beginning to the end of the NIHFOV, the end of NIHFOV means unplug the the connection of the non-invasive ventilation masks and a leaking valve to restore FB.

The software workstation MIM Masestro™ outlined the contours of the left, right lung each volunteer in the FB and in the NIHFOV state. The Maximum Range of Motion (MRM) of the diaphragm in the cephalic-caudal direction was recorded and analyzed. These included the MRM with FB (MRM_FB_) and the MRM with NIHFOV (MRM_NIHFOV_). The maximum density projection volumes of the left and right lungs in 4DCT in FB state were V_FB-L_ and V_FB-R_, and the maximum density projection volumes of the left and right lungs in NIHFOV state were V_NIHFOV-L_ and V_NIHFOV-R_. The left and right lung volumes (VL and VR) were recorded and analyzed. Coefficient of variation of lung volume = standard deviation of volume/mean volume. PtcO_2_, PtcCO_2_ and HR were recorded. The baseline values were the maximum PtcO_2_FB, PtcCO_2_FB, and HR.

### Statistical analysis

SPSS 22.0 statistical software was used for statistical analysis of the data in this study. Measurement data were expressed as mean ± standard deviation (SD), and t-test was used for comparative analysis, *P* < 0.05 was considered significant.

### Ethics statements

This study was approved by the ethics review committee of Wuwei Cancer Hospital. Written informed consent was obtained from participate.

## Results

### Subjective feedbacks

At the first training session, some subjects had mild suffocated breath, breathing fighting against the ventilator with multiple autonomous deep breaths. After communication with an experienced instructor, and with training session increased, breathing resistance were significantly reduced or disappeared after about 3–4 training sessions. According to the procedure comfort scale, 3 cases were excellent, 16 cases were very good, 4 cases were good. There were no “poor” or “not acceptable”. Some of the volunteers mentioned the noise of the air compressor and the vibration in the chest and abdomen caused by the high-frequency oscillation of the ventilator.

### Optimal ventilator parameter setting and duration of HFOV

All of the 23 volunteers (age range 22–69 years) completed the 7 sessions of HFOV training, The parameters for the entire group of subjects were: FiO_2_ (oxygen concentration) 100%, bias flow 40 L/min, RR 5–7 Hz, percentage of inspiratory time 33%, amplitude 5–7 cm H_2_O, average airway pressure 10 cm H_2_O, and the safety pressure alarm of the ventilator was set as 5–25 cm H_2_O.

Data of the last session of NIHFOV were obtained for analysis. The median duration of NIHPVO-on time was 32 min (range: 22–45).

### Data of maximum range of motion of diaphragm in the cephalic-caudal direction and variation of lung volume

**Figure 5:**
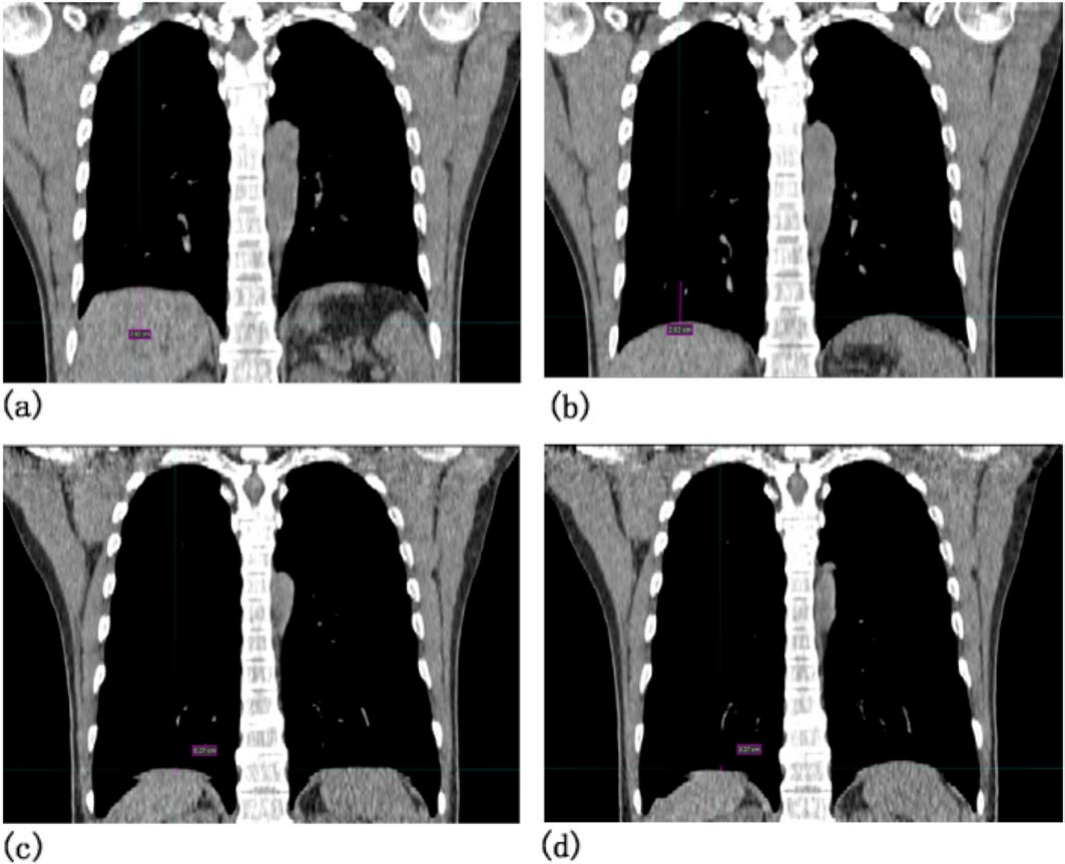
Comparison of the maximum movement changes in the diaphragm of a volunteer. (**a**) The minimum and (**b**) is the maximum position of the diaphragm with a maximum movement change of 2.82 cm in the cephalic-to-caudal direction under FB. (**c**, **d**) A maximum movement change of only 0.27 cm under NIHFOV.

Eight sets of 4DCT images under FB and 16 sets (8 subjects had 2 sets each) of 4DCT images with NIHFOV were obtained. A total of 24 CT images sets of bilateral lung and diaphragm were delineated. The MRM of the diaphragm, the volume of the maximum intensity projection of the left and right lungs (VL and VR) and the change of lung volumes with FB and NIHFOV were obtained, as shown in Tables 1 and 2, MRM_NIHFOV_ was significantly smaller than that in FB condition (1.8 cm vs 0.3 cm, *t* =  − 3.650, *P* = 0.003). Figure 5 shows a volunteer’s images of 4DCT under FB and NIHFOV, accord the images, we found the diaphragm with a maximum movement change in the cephalic-to-caudal direction were 2.82 cm under FB and 0.27 cm under NIHFOV.

**Table 1:** Maximum range of motion (MRM) of the diaphragm in the cephalic-caudal axis and lung volume.

	FB (mean ± SD)	NIHFOV (mean ± SD)	*t*	*P*
MRM of diaphragm (cm)	1.8 ± 1.3	0.3 ± 0.1	− 3.650	0.003
Left lung volume (ml)	1342.4 ± 242.5	1946.1 ± 180.7	4.238	0.012
Right lung volume (ml)	1689.8 ± 420.8	2107.2 ± 50.4	3.106	0.009

*FB* Free breathing, *NIHFOV* Non-invasive high-frequency oscillatory ventilation.

**Table 2:** Changes in medical physiological parameters with and without NIHFOV.

	FB (mean ± SD)	NIHFOV (mean ± SD)	*t*	*P*
PtcCO_2_ (mmHg)	36.5 ± 1.1	40.6 ± 0.5	17.676	< 0.001
PtcO_2_ (mmHg)	65.8 ± 4.6	340.7 ± 5.3	25.453	< 0.001
Heart rate (min)	72.5 ± 2.3	68.5 ± 3.1	1.257	0.233

*FB* Free breathing, *NIHFOV* Non-invasive high-frequency oscillatory ventilation, *PtcCO*_*2*_ Transcutaneous partial pressure of the CO_2_, *PtcO*_*2*_ Transcutaneous partial pressure of the O_2_.

All the volunteers completed the training sessions eventlessly, demonstrating a good tolerability of the procedure. The median NIHFOV-on time was 32 min (22–45 min). According to Table 1, the maximum range of motion (MRM) in the cephalic-caudal direction was significantly reduced on NIHFOV compared with FB (1.8 ± 0.8 cm vs 0.3 ± 0.1 cm, t =  − 3.650, *P* = 0.003).

The mean volume of the maximum intensity projection of the right lung was 1689.8 ± 420.8 ml with FB versus 2107.2 ± 50.4 ml with NIHFOV. Since NIHFOV was a positive-pressure ventilation, the right lung volume increased significantly compared with FB (t = 3.106, *P* = 0.009). The variation coefficient of right lung volume under NIHFOV was 0.024, and the change of right lung volume under NIHFOV was 50.4 ml, showing a good reproducibility. For the left lung, the variation coefficient of volume under NIHFOV was 0.092, and the change of volume under NIHFOV was ± 180.7 ml, indicating that the reproducibility was worse than that of the right lung. This may be caused by the volume variation of the stomach and the subsequent effect on the left lung.

### Changes in physiological parameters

The physiological parameters with FB and NIHFOV are shown in Table 2. According to Table 2, there is no statistically significant difference in heart rate between the two conditions (*t* = 1.257, *P* = 0.233). The median PtcCO_2_ increased by 4.1 mmHg (interquartile Range [IQR], 4–6 mmHg), a significant increase compared with FB (*t* = 25.453, *P* < 0.001). Both of the PtcCO_2_ with and without NIHFOV were within the normal range.

PtcO_2_ with NIHFOV increased significantly compared with FB (*t* = 17.676, *P* < 0.001). The PtcO_2_ of the subjects exceeded 200 mmHg about 3 min after the beginning of the training and continued to increase to over 300 mmHg, reaching a maximum of 369 mmHg. However, no symptoms or signs of oxygen poisoning were observed during the training. No hypercapnia was observed during the training .

## Discussion

### Significance of motion control

Respiratory movement leads to artifacts in the process of chest and abdomen imaging and limits the definition of magnetic resonance imaging and positron emission tomography-CT images^7^. With regard to radiotherapy, it increases radiation dose to the normal tissue adjacent to the target tumor. Consequently, the inhibition of respiratory movement during imaging capture and radiation therapy has been widely investigated^8^. The largest organ displacement caused by respiratory movement occurs in the cephalic-caudal direction. Table 3^9^ shows the mean, standard deviation and maximum displacement of some organs in these directions. According to the Report 76 of the American Association of Physicists in Medicine, the methods to reduce the influence of respiratory movement in radiotherapy can be divided into five categories: motion-encompassing, breath-holding, forced shallow breathing with abdominal compression, respiratory gating and real-time motion tracking^10^. These techniques have been widely used in clinical practice, and each method has its own advantages and disadvantages. Because of the growing popularity of stereotactic ablative radiotherapy (SABR)/SBRT and particle radiotherapy which have higher requirement for precision, the search for a feasible and affordable method of respiratory motion control has clinical significance^7^.

**Table 3:** Displacement (mm).

Organ	Average			Standard deviation			Maximum displacement in the direction of SI
	SI	AP	L	SI	AP	L	
Lung	11.8	4.7	3.2	12.6	2.3	2.1	50
Liver	25.6	–	–	14.5	–	–	55
Kidney	30	–	–	23.2	–	–	86
Pancreas	40.3	–	–	24.9	–	–	80
diaphragm	35.7	–	–	29.5	–	–	99

*SI* Superior–inferior, *AP* Anterior–posterior, *L* Lateral.

### Application of invasive and non-invasive ventilator-assisted breathing management

Ventilator-assisted respiratory movement control has been applied in radiotherapy worldwide. High-frequency ventilation (HFV) is one of this type of technique and has been reported to suppress thoracic movement under general anesthesia in patients with invasive ventilation in SABR and percutaneous radiofrequency ablation of tumors^11,12^. Most reported studies of ventilator-assisted radiotherapy used high-frequency jet ventilation (HFJV) and high-frequency percussion ventilation (HFPV) where general anesthesia was needed which made the whole procedure more complex with accompanying anesthesia-related risk. There are no reports of HFOV-assisted radiotherapy in the literature.

Functional apnea was applied for proton therapy in the Rinecker Proton Therapy Center (RPTC), Munich, Germany^13^. They reported that from September 2009 to November 2013, 61 patients received a total of 673 fractions of proton treatment. A total of 3025 apnea were performed: the anesthesia duration was 28–133 min (mean 57 min), each apnea time was 2.3 min (2–9 min), the proton therapy time was 30 min on average, and no adverse events occurred. Anesthesia recovery time ranged from 45 min to 3 h, with an average of 90 min. There were mild side effects such as throat irritation (1%), dysphagia (1%), hypotension (1%), nausea (1%) and drowsiness (3%). The mean target movement during apnea was 2 mm (range 0–4 mm). The technique uses endotracheal intubation under intravenous general anesthesia and mechanical ventilation. Mechanical ventilation was temporarily stopped during the proton beam-on period, while oxygen supply through the endotracheal intubation continued. During gantry rotation, the beam was off while mechanical ventilation was resumed. During the whole treatment, the patient’s pulse oxygen saturation was kept normal and the level of partial pressure of carbon dioxide in endexpiratory gas (P_ET_CO_2_) increased by 2–4 mmHg/min. Our center also applied functional apnea technique for radiotherapy in 2016^3^. Although it was effective on the management of respiratory motion, there were shortcomes of safety and logistic consideration.

Goldstein et al.^14^ reported their experience of movement management using continuous positive airway pressure (CPAP) ventilation in stereotactic lung radiotherapy. Under CPAP, the tumor motion in the superior-inferior, right-left and anterior–posterior plane decreases by 0.5–0.8 cm, 0.4–0.7 cm and 0.6–0.8 cm, respectively, and the lung capacity was significantly increased. Peguret et al.^15^ reported in 2015 percussion-assisted radiation therapy (PART) for managing respiratory movement. PART was initially tested in 10 volunteers and found to be well tolerated, allowing a median breath-hold time of 11.6 min (range 3.9 to 16.5 min). The technique was subsequently used in 3D conformal radiotherapy for breast cancer, SBRT for lung cancer and volumetric-modulated arc therapy (VMAT) for palliative pleural metastasis. The median breath-hold time was 11.6 min (3.9, 16.5) and the mean apnea-like breath-hold time was 7.61 min (SD = 2.3) without radiation interruption. Durham^8^ reported the PART used in Hodgkin lymphoma allows a marked reduction in heart dose.

Biro et al.^16^ reported in 2009 a study of liver movement inhibition in dogs under total intravenous anesthesia (TIVA) using high frequency jet ventilation (HFJV) with or without muscle relaxants. The results showed that the liver movement of anesthetized dogs could be controlled within 3.0 mm after the introduction of HFJV, and the injection of muscle relaxants did not further reduce the liver movement. Animal studies have shown that the use of HFJV can limit liver movement to a certain extent, and it can be used in human to reduce the amplitude of liver movement under TIVA to perform radiofrequency ablation of liver tumors^12^.

Non-invasive HFJV has also been reported. Ogna A et al. carried out trials of respiratory inhibition under high frequency ventilation on animals and human volunteers^17^. Prolonged apnea-like status could be achieved under NIHFJV with the respiratory frequency set at 250/min, resulting in a median duration of apnea of 20 min. And when the respiratory frequency was set at 500/min, the median duration of apnea was 5:16 min (3:57–6:48 min). The tidal volume of HFJV was dependent on respiratory rate and was 54 ml with a respiratory rate of 250/min and 26 ml with a rate of 500/min. PtcO_2_ was greater than or equal to 97%, and an increase of 6.2 mmHg of PtcCO_2_ was noted. Very small tidal volume results in a significant reduction in chest and abdominal movement compared to spontaneous breathing.

Management of respiratory movement with normal-frequency or high-frequency ventilator with or without sedation have been reported, but the procedures were relatively complex and had a high risk of hypercapnia, and the ventilator-on duration is not long enough for most precise radiotherapy.

### HFOV and NIHFOV

HFOV delivers small tidal volumes below the dead space volume with a high frequency of > 2 Hz breaths per minute, it can prevent ventilator-induced lung injury by decreasing the pressure of the airway, while providing adequate ventilation and oxygenation^4,5,18–20^ HFOV maintains alveolar inflation at a constant, less variable airway pressure with a sinusoidal air-flow oscillation to prevent the lung from the “inflate-deflate” cycle and provides improved oxygenation^18^. Because the lung is kept in a constant inflated and well-oxygenated status, the patient can only breathe very shallowly while maintaining a normal partial O_2_ level, thus creating the possibility of a minimum breathing amplitude with subsequent very small respiratory motion of the lung. HFOV can be supply by Care Fusion Oscillator 3100A and 3100B ventilatory and the Drager Babylog VN500 ventilator^5^. In the clinical, HFOV is a ventilation mode that can be used to protect the lung in a full spectrum of patients with acute lung injury, from neonates to adults^21^. HFOV is often used as a rescue strategy when conventional mechanical ventilation fails.

To our best knowledge, we are the first to report the application of NIHFOV for prolonged respiratory motion depression. The procedure, as tested by 23 healthy non-sedated adults, was smooth and feasible, and all of them had abundant blood oxygen without retention of carbon dioxide. NIHFOV may overcome some of the drawbacks of CPAP, PARP, and HFJV etc.

When we started the NIHFOV training, we chose the frequency of 250/min and the airway pressure of 15 cmH_2_O based on a literature review^18,19^. Although this setting could be tolerated, once the subject swallowed, the procedure would be interrupted due to high airway pressure. Thus we titrate and optimize them. At the end of seven training sessions, all the subjects could easily underwent more than 30 min without discomfort. In the study, we found the lung volume is increased with NIHFOV, The variation coefficient of right lung volume was 0.024, showing a good reproducibility. For the left lung, the variation coefficient of volume was 0.092, showed the reproducibility was worse than that of the right lung, after analysis, we found this was caused by the volume variation of the stomach and the subsequent effect on the left lung, we should pay particular attention in the practice.

In this study, we confirm the hypothesis that NIHFOV helps maintain normal blood oxygen and carbon dioxide levels, and allows a non-invasive and non-sedative approach of respiratory motion control. Compared with other means of methods, NIHFOV significantly prolonged apne-like duration.

At the time of this manuscript preparation, our team has successfully titrated the oxygen concentration down to 50%, and the longest HFOV training time was over 60 min; the PtcCO_2_ was still maintained at around 36 mmHg and the PtcO_2_ at 120 mmHg.

## Limitations

There are several limitations of this study. Pure oxygen inhalation was used in this experiment, and PtcO_2_ of the subjects reached more than 300 mmHg. This is equivalent to hyperbaric oxygen treatment which may affect the outcome of radiotherapy, and needs to be further studied. We chose pure oxygen based on the specification of the machine and the literature. But from our experience, pure oxygen may not be necessary for NIHFOV. Interestingly, the first author forgot to connect oxygen tube to the ventilator during one session of the training. Although only the room air was pumped, blood oxygen level was still within normal limit, and the training was also progressed normally, hinting that a reduced oxygen concentrations may still be workable with NIHFOV. We have started to examine the NIHFOV procedure with a lower oxygen concentration. We found when the subject was training with sitting position, saliva often appeared, some subjects experienced saliva accumulation after about 10 min in a sitting positive, which requiring inducing swallowing and causing uncomfortable and coughing feeling. This scenario did not occur with supine position. So the authors suggest volunteers to use supine position. There have been previous reports of complications such as dryness of upper respiratory tract and sore throat with using a noninvasive interface (HF-NIV)^17^. In this study, we kept the humidifier open and humidified the inhaled gas, avoided severe dryness and pain of upper respiratory tract.

Because 4-DCT has a 6-time higher exposure dose than that of conventional CT scanning^22,23^, we limited the application of 4DCT to only eight subjects. Because there were no tumor or metal markers, nor iodine oil as reference in the scanning areas, we used the diaphragm mobility in the cephalic-caudal direction as the endpoint of organ motion. However, it cannot measure precisely the mobility of the anterior–posterior and left-to-right direction. As this technique matures and is practiced in clinical, further study needs to be carried out in this respect.

This study systematically evaluated the effectiveness and safety of this technology, which enriched our team’s experience. We confirm that NIHFOV has good gas exchange function, longer apnea-like duration, low airway pressure with small tidal volume, no carbon dioxide retention occurs. At first, we also feel NIHFOV use is time-consuming, need a experienced technical team, but with most of our team members accepted the training as a healthy volunteers, we made a training video, summarize and exchange experiences, quickly make NIHFOV easy and acceptable. The subjective feelings of 23 volunteers were good, among whom the oldest volunteer was 69 years old, and all volunteers had fulfilled all training with good tolerance. The NIHFOV duration could easily exceed 30 min, and no CO_2_ retention ever occurred. In addition, in the implementation of this technique, like as HPJV and CPVP technique, the lung volume increases. In this study, it was found that the variation coefficient of the left lung was larger than that of the right lung. After analysis, we found that volume of air content of the gastric bubble was the reason why the variation of the left lung volume is obvious. It will affect the mobility and reproducibility of the left lung. This should be emphasized and avoided in practice.

This novel approach to managing respiratory movement using NIHFOV has never been previously reported. Our system is not a commercial system, and many parts are put together by our team. It took half a year for our team to learn how to use it harmoniously, because its efficacy is clear and its apply in patients is easily recognized and quickly mastered. In the initial stage of applying HFOV technology, we closely monitor all vital signs and various parameters, then we found that the technology is easy to operate, only a slight increase PtcCO_2_, there is no need to initial hyperventilation, for heart rate and blood pressure, there is no obvious fluctuations, the entire team mastered the technique, we simplify the process. All indexes and parameters of the subjects were monitored only during the training initial phase. Now we don't think it's necessary to measure blood pressure, or assess arrhythmias. Because it is an open system, the patient is not sedated, is sufficient oxygenated, and the training can be discontinued at any time during treatment. No side effects were observed. In the future, it will be applied in carbon ion therapy. Once finished carbon ion treatment and waiting for radiation attenuation session, patients can be informed through the microphone, and the connection can be unplugged and spontaneous ventilation can be resumed.

In conclusion, we demonstrate the safety, feasibility, and good tolerability of NIHFOV in suppressing respiratory movement in healthy volunteers. This study lays the foundation for further research on the potential benefits of this technique in improving the accuracy of carbon ion radiotherapy as well as photon radiotherapy, ensuring the accuracy of radiotherapy delivery, and further reducing the dose of normal tissues.

## Data Availability

The datasets generated and analyzed during the current study available from the corresponding author on reasonable request.

## References

[1] Yoganathan SA, Maria Das KJ, Agarwal A, Kumar S (2017). Magnitude, impact, and management of respiration-induced target motion in radiotherapy treatment: A comprehensive review. J. Med. Phys..

[2] Brandner ED, Chetty IJ, Giaddui TG, Xiao Y, Huq MS (2017). Motion anagement strategies and technical issues associated with stereotactic body radiotherapy of thoracic and upper abdominal tumors: A review from NRG oncology. Med. Phys..

[3] Dongji C, Ying Qi, Youguo Ma (2016). A case of right lung metastases hypofractionated radiotherapy under functional apnea. Chin. J. Radiol. Med. Prot..

[4] Johnson AH, Peacock JL, Greenough A (2002). High-frequency oscillatory ventilation for the prevention of chronic lung disease of prematurity. N. Engl. J. Med..

[5] Meyers M, Rodrigues N, Ari A (2019). High frequency oscillatory ventilation: A narrative review. Can. J. Respir. Therapy.

[6] Mizuno H (2019). Commissioning of a respiratory gating system involving a pressure sensor in carbon-ion scanning radiotherapy. J. Appl. Clin. Med. Phys..

[7] Nehmeh SA, Erdi YE (2008). Respiratory motion in positron emission tomography/ computed tomography: A review. Semin. Nucl. Med..

[8] Durham AD, Lovis A, Simons J (2020). Percussion assisted radiation therapy in Hodgkin lymphoma allows a marked reduction in heart dose. Radiother. Oncol..

[9] Alnowami MR, Hagi SK (2014). The battle against respiration-induced organ motion in external beam radiotherapy. Saudi Med. J..

[10] Keall PJ, Mageras GS, Balter JM (2006). The management of respiratory motion in radiation oncology report of AAPM Task Group 76. Med. Phys..

[11] Fritz P, Kraus HJ, Mühlnickel W (2010). High-frequency jet ventilation for complete target immobilization and reduction of planning target volume in stereotactic high ingle-dose irradiation of stage I non-small cell lung cancer and lung metastases. Int. J. Radiat. Oncol. Biol. Phys..

[12] Denys A, Lachenal Y, Duran R (2014). Use of high-frequency jet ventilation for percutaneous tumor ablation. Cardiovasc. Interv. Radiol..

[13] Eckermann M, Hillbrand M, Herbst M (2012). Scanning proton beam radiotherapy under functional apnea. Strahlenther. Onkol..

[14] Goldstein JD, Lawrence YR, Appel S (2015). Continuous positive airway pressure for motion management in stereotactic body radiation therapy to the lung: A controlled pilot study. Int. J. Radiat. Oncol. Biol. Phys..

[15] Péguret N, Ozsahin M, Zeverino M (2016). Apnea-like suppression of respiratory motion: First evaluation in radiotherapy. Radiother. Oncol..

[16] Biro P, Spahn DR, Pfammatter T (2009). High-frequency jet ventilation for minimizing breathing-related liver motion during percutaneous radiofrequency ablation of multiple hepatic tumours. Br. J. Anaesth..

[17] Ogna A, Bernasconi M, Belmondo B (2017). Prolonged apnea supported by high-frequency noninvasive ventilation: A pilot study. Am. J. Respir. Crit. Care Med..

[18] Hupp SR, Turner DA, Rehder KJ (2015). Is there still a role for high-frequency oscillatory ventilation in neonates, children and adults?. Expert Rev. Respir. Med..

[19] Vento G, Matassa PG, Ameglio F (2005). HFOV in premature neonates: Effects on pulmonary mechanics and epithelial lining fluid cytokines. A randomized controlled trial. Intensive Care Med..

[20] Yuan Y, Zhou L, Liu W, Dai Z (2021). Prospects and developments in the technologies of high frequency oscillatory ventilation. J. Biomed. Eng..

[21] Bouchut J-C, Godard J, Claris O (2004). High-frequency oscillatory ventilation. Anesthesiol. J. Am. Soc. Anesthesiol..

[22] Koste JR, Senan S, Kleynen CE (2006). Renal mobility during uncoached quiet respiration: An analysis of 4DCT scans. Int. J. Radiat. Oncol. Biol. Phys..

[23] Cover KS, Lagerwaard FJ, Senan S (2006). Color intensity projections: A rapid approach for evaluating four-dimensional CT scans in treatment planning. Int. J. Radiat. Oncol. Biol. Phys..

